# Comparison of the Efficacies of Dexmedetomidine and Clonidine in Preventing Postoperative Nausea and Vomiting in Paediatric Patients Undergoing Abdominal Surgeries

**DOI:** 10.7759/cureus.64789

**Published:** 2024-07-18

**Authors:** Khooshabo Gupta, Premraj Singh, Vinita Singh, Gyan Prakash Singh

**Affiliations:** 1 Department of Anaesthesiology and Critical Care, King George's Medical University, Lucknow, IND

**Keywords:** paediatric surgery, emergence agitation (ea), postoperative nausea and vomiting, clonidine, dexmedetomidine

## Abstract

Background

Postoperative nausea and vomiting (PONV) is a common complication in paediatric patients undergoing abdominal surgeries. Dexmedetomidine and clonidine, both α2-adrenergic agonists, have been proposed as potential treatments for PONV due to their antiemetic properties. This study aimed to compare the efficacies of dexmedetomidine and clonidine in the prevention of PONV in paediatric patients following abdominal surgeries

Methods

Eighty patients, aged five to 12 years undergoing abdominal surgeries under general anaesthesia were enrolled in this study. Patients were randomly assigned to receive either a single intravenous dose of 0.5 µg/kg of dexmedetomidine (Group D; n=40) or 1 µg/kg of clonidine (Group C; n = 40) 10 minutes before extubation. PONV was recorded for the next 24 hours.

Results

The baseline characteristics of patients were comparable. A higher proportion of patients in the clonidine group developed PONV as compared to the dexmedetomidine group (27.5% vs 20.0%, p=0.189). The visual analogue scale (VAS) score of group C was higher than that of group D (1.38±1.55 vs 1.00±1.26) but was not statistically significant. The Paediatric Anaesthesia Emergence Delirium (PAED) scale score or emergence agitation (EA) score was higher in group C during recovery time. The mean arterial pressures and the heart rates were comparable in both groups. No significant side effects were reported.

Conclusion

Our study concludes that dexmedetomidine and clonidine effectively control PONV in paediatric abdominal surgery, with no significant difference in incidence or severity. Dexmedetomidine significantly reduced PAED scale scores during recovery, indicating better control of EA. The two treatments showed comparable mean arterial pressures and heart rates without significant side effects.

## Introduction

Postoperative nausea and vomiting (PONV) is a typical unpleasant adverse effect following surgery that is sometimes dismissed as a minor side effect. However, PONV can worsen patient discomfort, lead to significant complications, and postpone patient discharge. PONV occurs twice as frequently in juvenile patients as it does in adults [1]. PONV is typically described as any occurrence of nausea, retching, or vomiting within the first 24 hours after surgery [2]. Its incidence in children is estimated to be between 33.2% and 82%, depending on risk factors [3]. The main risk factors for PONV or postoperative vomiting (POV) in paediatric anaesthesia are the duration of surgery (longer than 30 minutes), type of surgery (e.g., strabismus surgery), age > three years, use of an inhalational agent (e.g., nitrous oxide), previous PONV, and positive family history [1].

Dexmedetomidine is a powerful alpha-2 adrenergic agonist known for its wide-ranging effects, including anxiety reduction, sedation, pain relief, sympatholysis, and stabilization of hemodynamics, making it highly useful in clinical anaesthesia applications [4]. Using dexmedetomidine intraoperatively as an anaesthetic adjuvant has significantly decreased the need for opioids and inhalation anaesthetics. Clonidine is a significant sedative in anaesthesia and pain management. It is an alpha-2 adrenergic agonist medication that works more quickly and safely to reduce central and peripheral blood pressure than opioids and benzodiazepines do [5]. In order to examine the effectiveness of dexmedetomidine and clonidine as antiemetics in paediatric patients undergoing abdominal procedures, the current study was carried out. The primary objective of this study was to determine the incidence of postoperative nausea and vomiting in paediatric patients undergoing abdominal surgeries, comparing two distinct groups. Secondary objectives included evaluating the effects on postoperative emergence agitation (EA) using the PAED scale, assessing the effects on postoperative pain and the requirement for rescue analgesics within the first 24 hours postoperatively, and identifying any potential side effects or complications.

## Materials and methods

Children aged five to 12 years with American Society of Anesthesiologists (ASA) class I or II who were scheduled for abdominal surgery under general anaesthesia were enrolled in this clinical study after receiving approval from King George's Medical University (KGMU) Institutional Ethics Committee (approval no. V-PGTSC-IIA/P49). The trial was registered with the Clinical Trial Registry of India under the number CTRI/2022/09/045228.

Inclusion and exclusion criteria

Paediatric patients aged five to 12 years, of either sex, belonging to ASA class I or II, with body weight and height within ±20% of the ideal range, and scheduled for abdominal surgeries under general anaesthesia with a duration of 1 to 2 hours were included in the study.

Exclusion criteria for this study included children whose parents refused consent for their child's participation, as well as children with a known hypersensitivity to any of the study drugs. Additionally, patients who required a major surgery involving a blood transfusion, had a history of motion sickness, or were receiving antiemetic drugs before surgery were excluded. Moreover, patients who were on medications with antiemetic properties were also excluded from the study.

A routine preanesthetic check-up was conducted, and preoperative fasting was advised as per recommendation. On the day of the surgery, after taking the patient inside the operation theatre, appropriate-size Venflon (Becton, Dickinson and Company, Franklin Lakes, New Jersey, United States) was placed in the non-dominant hand, and lactated Ringer's solution was administered to all patients as per guidelines. Standard monitoring included an electrocardiograph (ECG), non-invasive blood pressure (NIBP), pulse oximeter, and capnography. Baseline hemodynamic parameters were recorded before the induction of anaesthesia. IV midazolam (0.01 mg/kg) was administered for premedication. Following preoxygenation, anaesthesia was induced with fentanyl (2 mcg/kg) and IV propofol (2 mg/kg). Once the patient was asleep, IV atracurium besylate (0.5 mg/kg) was given for endotracheal intubation. The endotracheal tube's correct placement was verified and secured. The lungs were ventilated using a 0.4 fraction of inspired oxygen combined with oxygen and nitrous oxide, employing volume control ventilation, and the ventilatory parameters were adjusted to maintain EtCO2 at approximately 30-35 mmHg. Anaesthesia was sustained with sevoflurane in 40% O2 and N2O, along with atracurium besylate. Fentanyl boluses and sevoflurane levels were adjusted to maintain the appropriate depth of anaesthesia.

Patients were randomly assigned to receive a single IV dose of either 0.5 µg/kg of dexmedetomidine (Group D) or 1 µg/kg of clonidine (Group C) 10 minutes prior to extubation. The randomization was done using a computer-generated random number table. We did not include a placebo control group, as it would be unethical given the risk of developing PONV following abdominal surgeries under general anaesthesia. The study drug was diluted with 0.9% normal saline to a total volume of 10 ml. The syringe containing the drug was prepared by an anaesthesiologist not involved in the study, while data collection was performed by a blinded anaesthesiologist. All patients were given a paracetamol infusion of 10 mg/kg for 20 minutes before the procedure ended. At the conclusion of the surgery, IV neostigmine (0.05 mg/kg) and glycopyrrolate (0.008 mg/kg) were administered to facilitate the return of spontaneous breathing, followed by endotracheal extubation. The patients were then transferred to the paediatric ICU (PICU) for further monitoring.

The scoring system for vomiting in the postoperative period (VPOP) is given in Table 1.

**Table 1:** Vomiting in the postoperative period (VPOP) scoring system Source: Bourdaud et al., 2014 [6]

Criteria	Points
Age	0 points = class 1: ≤ three years
	1 point = class 2: > three years and < six years or > 13 years
	2 points = class 3: ≥ six years and ≤ 13 years
Duration of anesthesia	0 points = ≤ 45 minutes
	1 point = > 45 minutes
Surgery at risk	1 point = tonsillectomy, tympanoplasty, strabismus surgery
	0 points = others
Multiple opioid doses	0 points = no
	1 point = yes
Risk percentage	
0 = 5%	0-1 = low risk
1 = 6%	
2 = 13%	2-3 = moderate risk
3 = 21%	
4 = 36%	> 4 = high risk
5 = 48%	
6 = 52%	

Statistical analysis

The incidence of PONV during the 24-hour period after surgery was the primary outcome measured in this study. The sample size consisted of 40 patients per group with 90% power and a Type 1 error of 0.05.

All statistical analysis was done using IBM SPSS Statistics for Windows, Version 21, (Released 2012; IBM Corp., Armonk, New York, United States). The values were represented in numbers (%) and mean±standard deviation (SD).

## Results

The demographic data of the patients included in the study are given in Table 2.

**Table 2:** Patients' demographic data SD: standard deviation

Parameter	Group D (dexmedetomidine, n=40)	Group C (clonidine, n=40)	p-value
Age (years, mean±SD)	6.99±1.61	6.45±1.14	0.088
Male/female (n%)	23/17 (57.5%/42.5%)	22/18 (55%/45%)	0.822

As given in Table 3, the VPOP score of paediatric patients enrolled in the study ranged from 2 to 5, and the median score was 3. Moderate VPOP scores (2-3) were observed in 41 patients, and severe VPOP scores (>4) were observed in the rest of the patients (39). Though severe VPOP scores were observed in a higher proportion of Group D patients compared to Group C patients (52.5%), this difference was not found to be statistically significant.

**Table 3:** Between-group comparison of VPOP scores VPOP: vomiting in the postoperative period

Sr. No.	VPOP score (risk)	Total (N=80)	Group D (n=40)		Group C (n=40)	
			No.	%	No.	%
1	Score 2-3 (moderate risk)	41	19	47.5	22	55.0
2	Score >4 (severe risk)	39	21	52.5	18	45.0
Mean VPOP±SD (range; median)		3.48±0.62 (2-5; 3)	3.45±0.50 (3-4; 4)		3.50±0.72 (2-5; 3)	

**Figure 1:**
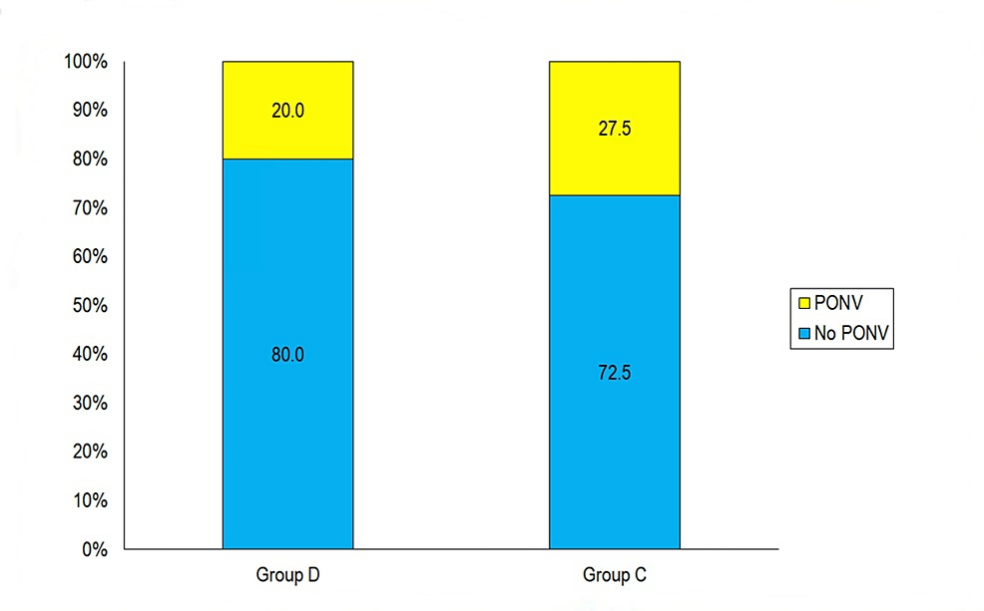
Between-group comparison of PONV incidences PONV: Postoperative nausea and vomiting

In our study, no incidence of PONV was observed in the majority of patients (n=61; 76.3%). The incidence of PONV was higher in Group C as compared to Group D (27.5% vs 20.0%) but this difference was not found to be significant statistically (Figure 1).

As given in Table 4, the range of the VAS scores (PONV) of the patients enrolled in the study was from 0 to 5. The mean VAS score was 1.19±1.42 (median: 1). The ranges (0-5) and median VAS scores (1) of both groups were similar. Though the mean VAS score of Group C was higher than that of Group D (1.38±1.55 vs 1.00±1.26), the difference was not found to be statistically significant. 

**Table 4:** Group comparison of VAS scores (PONV) VAS: visual analogue scale; PONV: postoperative nausea and vomiting; SD: standard deviation

Group	N%	Minimum	Maximum	Median	Mean	SD
Group D	50	0	5	1.0	1.00	1.26
Group C	50	0	5	1.0	1.38	1.55
Groups D and C combined	100	0	5	1.0	1.19	1.42

Postoperative analgesia was required for only 19 (23.8%) patients (Table 5). Though analgesic requirement was observed in a higher proportion of Group C patients compared to Group D patients (30.0% vs 17.5%), this difference was not found to be statistically significant. 

**Table 5:** Between-group comparison of postoperative analgesic use

Sr. No.	Analgesic use	Total (N=80)	Group D (n=40)		Group C (n=40)	
			N	%	N	%
1	Not required	61	33	82.5	28	70
2	Required	19	7	17.5	12	30

**Figure 2:**
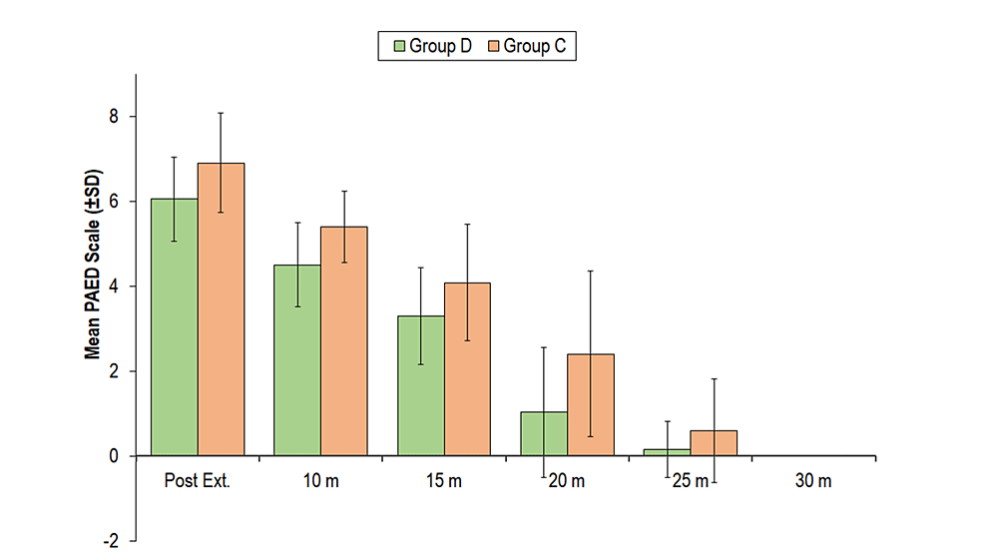
Group comparison of the PAED scale scores PAED: Paediatric Anaesthesia Emergence Delirium; SD: standard deviation; m: minutes; Post Ext: post extubation

The PAED scale score of Group C was higher as compared to Group D at all the above periods of observation except at 30 minutes. All the children (irrespective of the group) at 30 minutes had a PAED score of 0. None of the children had a PAED score >9 at any of the periods of observation (Figure 2).

## Discussion

PONV is a common adverse effect for patients undergoing general anaesthesia, especially in paediatric populations. The occurrence of PONV in children has been reported to be as high as 34%. Although there are several risk score models for adults, many are not applicable to paediatric patients [7]. Utilizing predictive risk scores to identify children at a higher risk for PONV can be beneficial in determining the most appropriate and cost-effective treatment strategies, potentially reducing hospital stay lengths and the frequency of unplanned admissions.

The purpose of this study was to compare the efficacies of dexmedetomidine and clonidine in preventing postoperative nausea and vomiting, their effect on postoperative pain, the requirements of rescue analgesics in the first 24 hours postoperatively in patients administered dexmedetomidine or clonidine, and their effect on postoperative EA by using the PAED scale. To our knowledge, this is the first study comparing the effects of dexmedetomidine and clonidine on postoperative nausea and vomiting in paediatric patients who underwent abdominal surgeries.

The participants in the study ranged in age from five to 12 years. The average age in the dexmedetomidine group was 6.99±1.41 years, while in the clonidine group, it was 6.45±1.14 years; this difference was not statistically significant. Most of the participants were male (57.5% in the dexmedetomidine group and 55% in the clonidine group), and the male-to-female ratio was similar across both groups. The majority of the patients were classified as ASA-I. These factors indicate that the groups were comparable and that the randomization process was effective.

To aid in the prevention of paediatric PONV, Bourdaud et al. [6] created a risk-predictive scoring model for postoperative vomiting, known as the VPOP scoring system. In order to determine the severity of the risk, the generated score stratifies five independent risk factors for vomiting in children. These include age, duration of anaesthesia, surgery at risk, a propensity to postoperative vomiting, and multiple opioid doses. The scores range from 0-6, with 6 representing children at the highest risk. In our study, the VPOP scores ranged from 2 to 5, and the median score was 3. Moderate scores (2-3) were observed in 41 children, and in the rest of the 39 patients, severe VPOP scores (>4) were observed. Though severe VPOP scores were observed in a higher proportion of group D patients compared to group C patients (52.5%), this difference was not found to be statistically significant.

Dexmedetomidine offers perioperative hemodynamic stability, effective anaesthesia, an opioid-sparing effect, a reduction in PONV, and control of postoperative pain, all with minimal respiratory depression [8-9]. A meta-analysis by Liang et al. related to clinical uses of dexmedetomidine for PONV prevention reported that a 0.5-1.0 mcg/kg bolus infusion was effective in reducing the occurrence of PONV [10].

In our study, the dexmedetomidine group showed better control of PONV than the clonidine group during the first 24 hours after the surgery. The incidence of PONV was higher in the clonidine group compared to the dexmedetomidine group (27.5% vs 20.0%), but this difference was not found to be statistically significant. The VAS scores (PONV) of the patients enrolled in the study ranged from 0 to 5. The mean score was 1.19±1.42 (median: 1). The range (0-5) and median VAS score (1) of both groups were similar. Though the mean VAS score of group C was higher than that of group D (1.38±1.55 vs 1.00±1.26), this difference was not found to be significant statistically. These results may be attributed to reduced noradrenergic activity due to alpha-2 presynaptic inhibition in the locus coeruleus or a decrease in sympathetic outflow, which can trigger PONV [11]. 

Our findings are consistent with the study of Li et al., who demonstrated that the administration of different doses of dexmedetomidine (0.3µg/kg vs 0.5µg/kg) in paediatric patients undergoing strabismus surgery reduced the incidence of PONV [1]. A study by Chiang et al. found that dexmedetomidine use reduced PONV in paediatric patients scheduled for strabismus surgery [12].

Moreover, Gupta et al. demonstrated that intraoperative administration of dexmedetomidine (1 µg/kg loading dose followed by a 0.5 µg/kg/h infusion) in children undergoing spinal surgery reduced the incidence of PONV [2]. Chen et al. also reported that intraoperative dexmedetomidine (1 µg/kg loading dose followed by a 0.5 µg/kg/h infusion) decreased postoperative vomiting in paediatric patients undergoing strabismus surgery [13]. Compared to these previous studies, the effective dose in our study was relatively low (0.5 µg/kg). This may be related to the different procedures done, the length of the surgery or anaesthetic, the age of the patients who were recruited, and the combination of anaesthetic medicines used to calculate the dose necessary to effectively lower the incidence of PONV.

Clonidine is less expensive and has fewer side effects than other medications like opioids and benzodiazepines; it has been suggested as a useful treatment for the preoperative period. Clonidine is an alpha-2 agonist used as a sedative premedication in children [14]. It has been demonstrated that this medication can lessen the need for anaesthesia, offer prior sedation, and inhibit the cardiovascular response to tracheal intubation and laryngoscopy. Additionally, clonidine has been proven to decrease the incidence of PONV following systemic and epidural administration, and it has been reported to lessen shivering following both general and spinal anaesthesia [15-16].

However, the published literature shows conflicting results regarding clonidine's impact on the incidence of PONV. Some studies in adults have found that oral clonidine can decrease the occurrence of PONV [17-18]. In the present study, 1 µg/kg of intravenously administered clonidine reduced the occurrence of PONV during the first 24 hours after surgery.

Our results are consistent with Mikawa et al., who demonstrated that preoperative oral clonidine at a dose of 4 µg/kg is associated with a reduced incidence of postoperative vomiting in children undergoing strabismus surgery [19]. In addition, Alizadeh et al. found that administering oral clonidine at a dose of 4 µg/kg before surgery is linked to a lower incidence of postoperative vomiting in children who have had an appendectomy [20]

EA is a common adverse postoperative complication in children. The possible risk factors of EA include rapid emergence from anaesthesia, the use of short-acting volatile anaesthetic agents, postoperative pain, age, and surgery type [21]. Many scales have been designed to recognize the severity of EA. In the PAED scale, five listed behaviours (eye contact, purposeful movement, awareness of surroundings, restlessness, and inconsolability) are added to achieve a total score of 20 (the maximum score of 20) [22]. Children with a PAED score of 10 or higher, or 12 or higher, were diagnosed with EA.

In our study, PAED scores for EA were lower in the dexmedetomidine group compared to the clonidine group during all the periods of observation except at 30 minutes. All the children in both groups at 30 minutes had a PAED score of 0. None of the children had PAED scores >9 at any period of observation.

Our findings are consistent with those of John et al., who demonstrated that rapid IV bolus (0.5 µg/kg) administration of dexmedetomidine in children reduces EA without causing hemodynamic instability [23]. In addition, Cho et al. demonstrated that in children who underwent adenotonsillectomy, perioperative dexmedetomidine administration was associated with a reduction in the incidence of EA [24].

In this study, postoperative analgesia was required in only 19 patients (23.8%). Although postoperative pain was less in the dexmedetomidine group and rescue analgesic requirements were lower in the dexmedetomidine group compared to the clonidine group (17.5% vs 30%), this difference was not found to be significant statistically.

Our observation of decreased postoperative pain is consistent with Gurbet et al.'s work, which demonstrated that dexmedetomidine infusions (1 µg/kg loading dose over 10 minutes, followed by 0.5 µg/kg/h) during abdominal surgeries provided effective postoperative analgesia without increasing side effects [25]. This reduction in postoperative pain may be due to the inhibition of substance P release from the dorsal horn via activation of alpha-2 adrenoreceptors, resulting in decreased nociceptive input. In addition, Olutoye et al. showed that giving dexmedetomidine 1 µg/kg to kids undergoing adenotonsillectomy had the benefit of extending the time until the first analgesic and reducing the requirement for rescue analgesia after surgery [26].

In terms of hemodynamic stability, dexmedetomidine and clonidine both can cause a decrease in blood pressure (BP) and heart rate (HR) by reducing the sympathetic outflow and circulating catecholamines associated with higher doses of dexmedetomidine and clonidine.

In our study, there was no significant bradycardia or hypotension observed during the observation period. The HRs and mean arterial pressures (MAPs) in both the dexmedetomidine and the clonidine groups were comparable throughout the period of observation.

Limitations

Our study has several potential limitations that should be noted. Firstly, the sample size was relatively small, which may limit the generalizability of the findings. Secondly, the study was conducted at a single centre, which may introduce bias and limit the applicability of the results to other settings. Finally, the follow-up period was relatively short, which may not capture long-term outcomes and effects. These limitations suggest the need for further research with larger, multi-centre studies and longer follow-up periods to validate and expand upon our findings.

## Conclusions

Based on our study, we conclude that both dexmedetomidine and clonidine are effective in controlling PONV in paediatric patients undergoing abdominal surgery, with no significant difference in the incidence or severity of PONV between the two groups, as the VAS score did not show statistical significance. However, dexmedetomidine demonstrated a statistically significant reduction in the PAED scale during recovery, indicating better control of EA compared to clonidine. Although the PAED scale was a secondary outcome and thus not conclusive, it suggests that dexmedetomidine may offer additional benefits in managing emergence agitation. Both treatments resulted in comparable MAPs and HRs, with no significant side effects reported. These findings suggest that while both medications are effective for PONV management, dexmedetomidine may be superior for controlling EA.
